# Implementation of the Family HELP Protocol: A Feasibility Project for a West Texas ICU

**DOI:** 10.3390/healthcare9020146

**Published:** 2021-02-02

**Authors:** Rebecca McClay

**Affiliations:** School of Science, Technology, Engineering, and Math, American Public University System, Charles Town, WV 25414, USA; rebecca.mcclay@gmail.com

**Keywords:** family HELP, hospital elder life program, bedside nurse, delirium, critical care, ICU

## Abstract

The purpose of this project was to determine if bedside intensive care unit (ICU) nurse buy-in to the Family Hospital Elder Life Program (HELP) protocol was sufficient to make implementation feasible at one county hospital in West Texas. Surveys were anonymous with ballot box collection being available to the bedside ICU nurses for one week each. Questions were based on literature findings of expected outcomes, identified barriers and facilitators, Calgary Family Intervention Method framework domains, and the Centers for Disease Control and Prevention Framework for program evaluation. Outcome measures were taken from the stated aims of the project and evaluated from paired baseline and summative survey questions. Survey participation was approximately half of nurses employed in the studied ICU. Analysis of the surveys showed a positive perception of family presence decreasing patient delirium symptoms, and a positive perception of the Family HELP protocol. The results described a high perception of family members as partners in care and high intention to implement the Family HELP protocol, indicating strong support of a full implementation of the protocol. The high level of bedside nurse buy-in present in this study has large implications for successful implementation of the Family HELP protocol in the near future, with sustainability and continued use supported by potential inclusion of the task in the electronic health record charting.

## 1. Introduction

Decreasing delirium occurrence and severity in the intensive care unit (ICU) is a significant concern and high priority for the Society of Critical Care Medicine (SCCM), an international interdisciplinary group that sets ICU clinical practice guidelines, and the American Geriatrics Society (AGS) [1,2]. The Hospital Elder Life Program (HELP) is a set of comprehensive patient centered guidelines providing optimal care for hospitalized older persons that has recently been folded into the AGS CoCare portfolio [2]. The HELP program is broken into smaller implementable protocols, facilitating introduction to the larger program [3]. Family HELP is one such protocol, which has demonstrated reduced delirium in older adults through inclusion of family members into the healthcare team [4]. The AGS and the SCCM promote including family engagement as a means of reducing ICU delirium, but there is little published about bedside nurse acceptance of implementing protocols that support bedside family education.

### 1.1. Problem Description

The selected West Texas hospital did not have a delirium protocol in place. They had a perceived high rate of discharge to long term care rather home, and bedside ICU nurse burnout was identified as influenced by increased burden of care for patients with delirium symptoms. Their ICU is an open unit with full visitation privileges, where most patients had prolonged in room family visitation. However, while these family members were considered part of the patient care team, they were rarely engaged in formal therapeutic interventions. 

Informal gap analysis showed lack of ICU nurse awareness that evidence-based nonpharmacological interventions can lessen or shorten ICU delirium. Lack of a family delirium education protocol was identified as the root barrier to inconsistent bedside teaching through root cause analysis from a group of the hospital bedside ICU nurses using the “Five why” approach [5]. The Family HELP protocol was selected to address this through posted interventions supporting consistent and methodical nurse-to-family teaching and reinforcing family knowledge of appropriate interactions to reduce delirium [6]. Nurse willingness to participate and an open family visitation policy were identified as significant facilitators to this intervention [7,8]. It was determined that nurse acceptance of a structured protocol supporting nurse-to-family delirium and intervention education should be explored.

### 1.2. Available Knowledge

Importance of nurse-to-family delirium reduction intervention education is best understood through the impact of ICU delirium on patient and family units. Family involvement and nurse buy-in are essential to the implementation and acceptance of the validated Family HELP protocol. Current literature was evaluated for these components with the inclusion of sentinel and directly relevant articles.

Impact of delirium: Delirium is an acute organ failure of the brain creating a state of confusion and biologic instability known to be as high as 80% in mechanically ventilated patients, up to 87% of ICU admissions, and appears to be even higher in rural and indigenous populations [3]. The cost estimate of ICU delirium ranges from $4 to $16 billion annually in the United States, but impact goes beyond financial cost to patient and family quality of life to include significant nursing emotional and physical burden [8]. Direct implications include hemodynamic and respiratory instability, substantial functional decline, longer ICU stays, longer mechanical ventilation dependence, and higher morbidity and mortality rates with secondary complications including falls, bedsores, and hospital acquired infections [8,9]. After discharge, ICU patients diagnosed with delirium are 2.5 times more likely to be discharged to skilled nursing facilities rather than home, and patients can require months or years to overcome effects including Post Intensive Care Syndrome [10]. Extended impacts lasting years include adverse sequalae of physical, psychological, and financial effects increasing patient and family burden from loss of income and decreased quality of life [11]. With the burden of delirium creating a high cost to individuals, families, and stakeholders, efficient and effective reduction is essential [4,12]. 

Family involvement: Family health is decreased when members are hospitalized, making inclusion in treatment important to the family dynamic as well as patient outcomes [7]. The role of family and empowerment in patient care is an area needing expansion in the ICU, and was included in the SCCM 2016 guidelines [1,6]. Family members providing directed nonpharmacological interventions reduces delirium symptom severity, duration, and need for antipsychotic medications [12,13]. 

Nurse buy-in: Nurse buy-in to early delirium intervention improves patient outcomes and nurse compliance [1,7]. Family HELP guided family education on the importance and proven effectiveness of specific actions in decreasing delirium has been highly successful in motivating behavioral and belief changes in families [14]. Bedside nurse teaching is an integral part of protocol success, as families prefer one-on-one education specific to their family member and nurse delivered education opens communication between ICU staff and family members [7,12]. Acknowledgement as partners increases family contributions, feelings of respect, and preparation for discharge [7]. Introducing Family-HELP as co-operative care provides nurses a framework to encourage structured and meaningful family interaction with critically ill patients while reducing nurse task burden of keeping agitated patients safe from self-harm and exposure to delirium induced aggression [6,8,15]. 

Family HELP: The Family HELP protocol, a section of the larger HELP guidelines, is a highly efficient and cost-effective program that facilitates nurse-to-family teaching of nonpharmacological interventions which effectively decrease delirium duration, severity, and length of stay [2,4,11,14]. The protocol includes written information to standardize, guide, and reinforce consistent nurse-to-family education on delirium and beneficial tasks relative to patient care and family dynamics [4,14]. Family HELP engages directed family-to-patient specific intervention activities that increase family empowerment and improve patient and family function, increasing caregiver satisfaction with patient care and outcomes [16,17]. 

### 1.3. Rationale 

The Calgary Family Intervention Model (CFIM) was chosen to guide the rationale for this project based on documented use in HELP and Family HELP implementation studies and to facilitate implementation if warranted by the feasibility study [6,7,14]. CFIM is family centered and supports normal capacities of family members, as much as possible, while the patient is admitted to the hospital through empowerment and improved respectful communications of care options [18,19]. Each aspect of the Family HELP protocol was evaluated for fit into the cognitive, behavioral, and affective domains of the CFIM (Table 1) [19]. Using the CFIM domains as the focus of implementing the Family HELP protocol supports nurse influence on personalizing care to the family and demonstrates the effectiveness of nurse effort to include families in patient care [6]. 

### 1.4. Aims

Purpose. The purpose of the project was to determine if there is sufficient bedside ICU nurse buy-in to the Family HELP protocol to make implementation feasible at one county hospital in West Texas. 

Aim 1: Obtain test unit nurse baseline perceptions of caring for patients with delirium and the impact of family interventions. 

**Figure 1:**
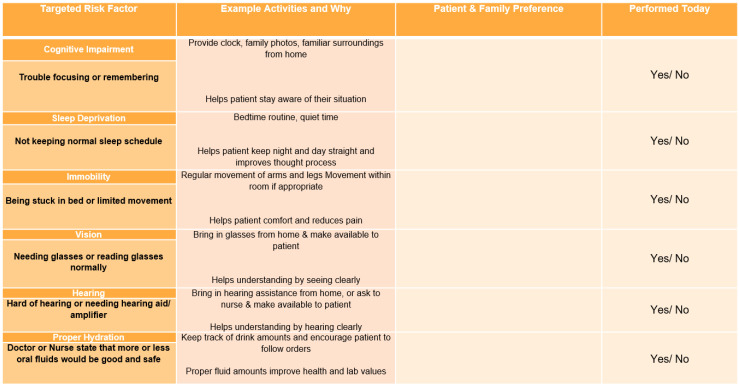
Family Hospital Elder Life Program (HELP) in room poster as posted in patient rooms and implemented at the site for bedside nurse-to-family education. Poster was printed as an 11” by 14” laminated poster.

Aim 2: Complete in person education of ICU bedside nurses on the evidence-based Family HELP protocol effectiveness and proper implementation with poster (Figure 1). 

Aim 3: Obtain summative nurse perceptions of the Family HELP protocol tool, family interactions at the bedside, and factors impacting sustainability.

## 2. Materials and Methods 

### 2.1. Context

The test ICU was in a not-for-profit county hospital in West Texas, serving an urban city as well as surrounding rural and frontier classified counties. The hospital has approximately 475 beds, of which 25 are dedicated mixed ICU beds with 43 ICU nurses. While the hospital typically maintains an open visitation policy allowing family around the clock presence, COVID 19 safety measures eliminated ICU visitation during this project. Survey exclusion criteria was by staff choice to not participate. Elements identified to most likely to support Family HELP protocol buy-in were full support of the Unit Director and Clinical Managers, and a hospital culture promoting nurse driven change. Potential barriers included bedside nurse refusal or perceived time requirement to participate.

### 2.2. Intervention

This feasibility project was to determine the support level and perception of the Family HELP protocol by bedside ICU nurses for future implementation. This was accomplished through baseline and summative nurse perception surveys bracketing an introduction to the Family HELP protocol and a 1-week opportunity to implement it with family members.

The first step was adapting the Family HELP poster from the Rosenbloom-Brunton et al. [6] work to the hospital with plain language and then sending it for printing. Before any teaching or poster placement occurred, a baseline anonymous paper-based 5-point ordinal survey with ballot box collection was available to bedside ICU nurses in their break room for 7 days. Full project disclosure was the first page of each survey. Fliers inviting participation were posted in staff only areas of the unit during this time. After surveys were removed, bedside nurse education on the Family HELP protocol was performed by the primary investigator during day and night shift huddles with tracking of education by employee list. The education included outcomes of Family HELP use in other facilities, poster orientation, how to introduce the poster to family members, and optional family use of the laminated section if they choose to document intervention information. After education was complete for all ICU nurses and clinical managers, laminated posters were posted in all ICU rooms using damage free adhesive strips. 

One week after poster placement family visitation was no longer allowed for most ICU patients due to the COVID-19 pandemic. In-room posters remained in place allowing nurses to utilize the content for bedside education of patients, and phone education of family members. At 6 weeks after poster placement, a nonmatched summative survey was made available using the same format, availability, and flier invitation locations as the baseline survey. Surveys were collected after 7 days, and data from both surveys was recorded in a spreadsheet for conversion to a STATA data file.

### 2.3. Study of Intervention

The Centers for Disease Control and Prevention (CDC) Framework for program evaluation in public health was used to guide the process of evaluation for this project, and standards of effective evaluation were upheld throughout the evaluation. Feasibility of the intervention required analysis of nurse buy-in to the Family HELP protocol and facilitators that would support sustainability. Propriety of legal, ethical, and due regard of participant welfare was assured by the project evaluation by the hospital Internal Review Board (IRB), and accuracy of outcome measures is assured by reporting raw and analyzed data for evaluation of the reader. 

### 2.4. Measures

Questions on the baseline and summative bedside ICU nurse perception surveys were based on literature findings of expected outcomes, identified barriers and facilitators, and CFIM framework domains. Each of the outcome measures was taken from the stated aims, and are presented with definitions, significance, and reason for evaluation.

Family education relevance to job of the nurse—Assessment of ICU bedside nurse perception of nurse-to-family effect on the cognitive domain [19]. 

Caring for patients with delirium—Perceived burden of caring for patients with delirium has been shown to be elevated in bedside nurses [9,20]. 

Impact of intervention education: Family benefit—Increasing meaningful family interactions is one of the anticipated outcomes of using the Family HELP protocols [12].

Impact of intervention education: Patient benefit—Family HELP interventions have demonstrated improved patient outcomes [4].

Benefit of Family HELP in-room poster—The poster reinforces the cognitive domain and supports family inclusion of taught intervention behaviors [18].

Perceptions of the Family HELP protocol tool—Nurse perception of usefulness, as well as intent to use, are important to evaluating fidelity to the protocol and feasibility of implementation [11].

Nurse perceptions of family at bedside—Family members are often considered an important part of the medical team promoting inclusion and education [15]. 

Understanding and intent to implement—Implementing a protocol with diligence requires cognitive and affective inclusion by nurses providing the education [19]. 

Factors that may or may not increase their likelihood implement the tool—Cognitive acceptance of the Family HELP tool may not translate into a behavioral change, so understanding components that can support behavioral inclusion of the Family HELP teaching is important to creating and sustaining behavioral change [18]. The study hospital electronic health record charting had two team identified prompts to promote actions by the ICU nursing staff, timed tasks added to the Care Compass view, and interdisciplinary plans of care (IPOC) task lists.

Data completeness was ensured through the inclusion of all survey results. There was a total of 43 nurses employed in the ICU at the time the project was initiated. The number of baseline initial surveys was 22 = 51% response rate. The number of summative surveys was 21 = 48% response rate. Validity of the survey was anticipated through expected measures found in the literature, such as care burden and perception of family role, to be reflected in the survey instrument used for this project. Due to novel use, reliability of survey questions was not measured directly but may become measurable if full implementation includes used questions. 

### 2.5. Analysis 

Analysis of positive responses (score of 4 or 5) for each measure are provided in results with the survey question groupings, and their outcomes. Descriptive statistics of mean and percentage agreement from the surveys were used.

### 2.6. Ethics

Considerations. While the feasibility project did not pose an ethical dilemma and there are no known risks for participation in the Family HELP protocols, ethical considerations must be evaluated to protect the human subjects [4]. Benefits to the nursing staff include the improved perception of the burden of care for patients experiencing ICU delirium, and educating family member on appropriate family driven actions could reduce patient delirium symptoms [9,20,21].

Respect for persons. Nurses maintained their autonomy and were protected from exploitation by maintaining the choice to participate at any time [21]. 

Beneficence. Disclosure information shared was with the autonomous participants as the first page of the survey, while open and frank feedback on perceptions and experience was encouraged by providing open ended space on the surveys [21]. 

Justice. Potential risk of unanticipated side effects was low as there are not currently any known major potential risks to anonymous and voluntary perception surveys, and the poster was a furnishing in the room with no formal recording of whether teaching occurred [12]. 

Ethics approval. The study hospital IRB designated the project a quality improvement study that did not require IRB oversight. 

## 3. Results

Approximately half of nurses employed in the studied ICU participated in the surveys, and analysis of responses demonstrated a positive perception of family presence at the bedside and the Family HELP protocol use. Over the course of this project there was a significant change in family visitation policies secondary to COVID-19 which potentially influenced the nursing opinions on the summative survey. 

Family education relevance to job of the nurse—The baseline question, “How relevant and helpful do you think family education on delirium is for your job?” 88% (4.4 out of 5) was matched with the summative question, “How relevant and helpful do you think educating family members on delirium is to your job?” 90% (4.5 out of 5).

**Figure 2:**
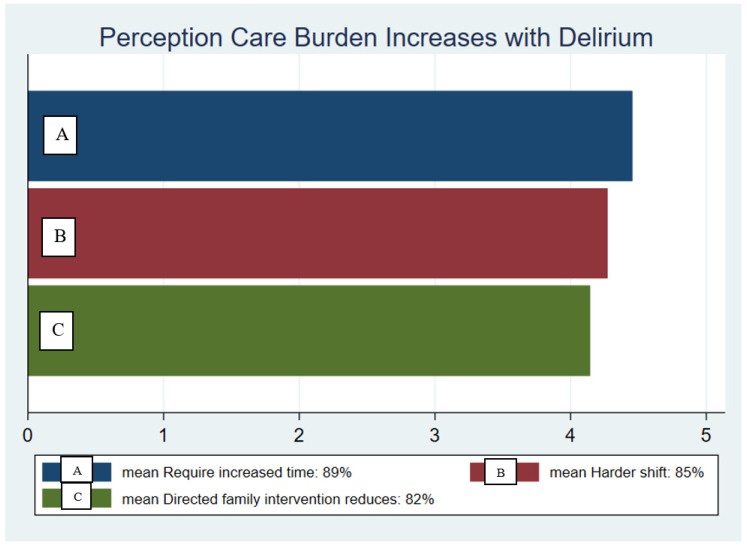
Nurses’ perceived burden of care when patients experience delirium at baseline, as measured by the percentage of surveyed nurses that believe there is an increased amount of time required to provide care (**A**) and believe that their shift is “harder” when caring for patients with delirium symptoms (**B**). The bottom bar indicates the percentage of surveyed nurses that believe directed family interventions reduce the nursing burden of care for patients experiencing delirium (**C**).

Caring for patients with delirium (Figure 2)—Two questions from baseline survey, “Do you feel that patients experiencing ICU delirium take more of your time to care for and keep safe?”89% (4.45 out of 5) and “Does caring for a patient with delirium make your job as a nurse harder than caring for a patient that is not experiencing delirium?” 85% (4.27 out of 5), and summative survey question, “Do you feel directed family interventions, such as those outlined on Family HELP posters, would lessen your workload for patients with delirium?” 82% (4.14 out of 5). 

Impact of intervention education: Family benefit—The baseline question, “Do you feel families would benefit from family education on delirium reduction methods?” (4.72 of 5 = 94%) was compared to the summative question, “Do you feel the families benefit from being taught focused interventions for their loved ones?” (4.81 of 5 = 96%). 

Impact of family interventions on patients: Patient benefit—Baseline survey question, “Do you feel your patients would benefit from family education on delirium reduction methods?” (4.27 of 5 = 85%) paired with the summative survey question, “Do you feel that family–patient interactions are more beneficial when family members are taught patient medical and sensory needs?” (4.68 of 5 = 94%).

Benefit of Family HELP in-room poster—Benefit of use was assessed by the baseline question, “Do you feel a written reminder to families on contributions they can make to lessen delirium would be useful?” (4.27 of 5 = 85%) paired to the summative question, “Do you feel the Family HELP in-room poster will support family interventions presented during the nurse-to-family education?” (4.57 of 5 = 91%).

**Figure 3:**
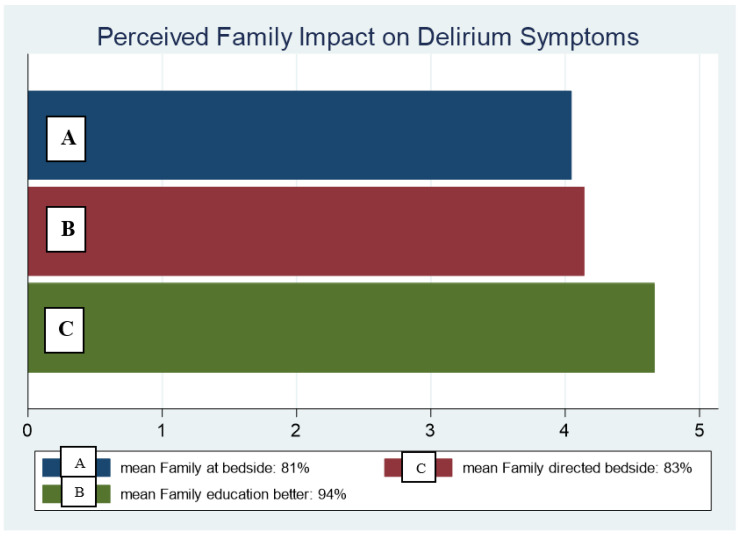
Nurse perceptions of family presence at bedside with the belief that family presence at the bedside without directed interventions decreases delirium symptoms, compared to nurse directed family interventions, and Family HELP directed interventions after bedside nurse-to-family education.

Nurse perceptions of family at bedside (Figure 3)—The baseline question, “Do you feel patients suffer less delirium symptoms when family members are at the bedside?” 81% (4.04 out of 5) was paired with the summative questions, “Do you feel directed family interventions, such as those outlined on Family HELP posters, would lessen your workload for patients with delirium?” 83% (4.14 out of 5) and “Do you feel that family–patient interactions are more beneficial when family members are taught patient medical and sensory needs?” which resulted a score of 94% (4.67 out of 5). 

Understanding and intent to implement—The perceived ability to implement was measured by the summative question, “Does the Family HELP tool feel easy to teach?” 90.4% (4.52 out of 5). Intent to implement was assessed in the summative question, “Do you feel you will likely use the Family HELP poster to educate family members?” 90.4% (4.52 out of 5). 

**Figure 4:**
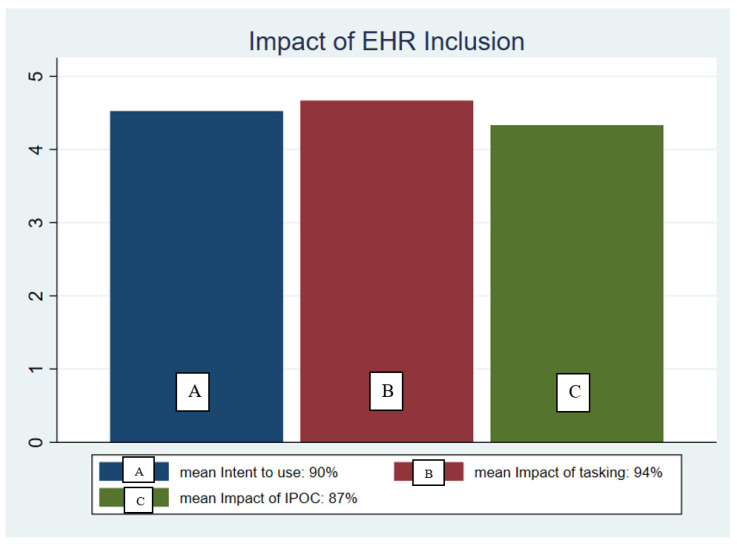
Nurse perceptions of electronic health record charting factors that may influence the likelihood of nurses consistently implementing the Family HELP tool.

Factors that may or may not increase their likelihood implement the tool (Figure 4)—The intent to use the protocol (4.52 of 5 = 90%) was compared to summative survey questions, “Would having the Family HELP teaching task added to your Care Compass increase your use of the tool when family members are allowed visitation again?” (4.67 of 5 = 94%) and “Would having a Family HELP protocol included in an IPOC such as “Knowledge deficit” available increase your satisfaction with the tool?” (4.33 of 5 = 87%). 

## 4. Discussion

### 4.1. Summary

Perceptions of the Family HELP protocol aligned with previous studies pertaining to nursing perceptions of delirium care and family inclusion. This study revealed overall positive perceptions and a high intent to use the protocol, demonstrating validity of the study purpose of having sufficient bedside ICU nurse buy-in to the Family HELP protocol to make implementation feasible within the studied hospital unit. Most nurses that responded indicated that they would use the Family HELP protocol and in-room poster to provide nurse-to-family education on delirium and appropriate nonpharmacological interventions shown to reduce delirium symptoms. Most bedside ICU nurses believed that educating family members about delirium was appropriate to their role. There was strong belief that family members performing relevant interventions would lessen the patient care burden associated with caring for and protecting patients with delirium and that both families and patients would benefit from the Family HELP teaching. Intent to use increased with inclusion of a Care Compass timed task but was negatively associated with inclusion as an IPOC item within the electronic health record. 

### 4.2. Interpretation

Results describing family members as partners in care and implementation outcomes of the Family HELP protocol are in line with the literature, indicating strong support of a full implementation of the protocol. The positive correlation with intended compliance and assigned timed task, as well as the negative correlation with making it an IPOC element should be considered at full implementation. The reason IPOC documentation was considered unfavorable is unclear and warrants exploration before full implementation. The education of bedside ICU nurses appears to have been effective, with a high level of confidence in nurse-to-family teaching of the content. Adding Family HELP in-room poster importance during bedside nurse orientation warrants consideration, as their belief in poster importance to reinforcement is less positive than the literature indicates. 

### 4.3. Limitations 

Generalizability of this project is limited, as the sample population was specific to ICU nurses at a single hospital. The complexity of the COVID-19 pandemic and little family visitation limited nurse ability to implement the protocol. Moreover, it was not possible to test the inclusion of the Family HELP teaching into the electronic health record for impact. 

## 5. Conclusions

The increase in aging population and expansion of the need for intense medical intervention means that more people are spending time in intensive care units of the hospital. The high occurrence rate leading to high cost to manage, treat, and recover from ICU delirium makes lessening symptoms and duration an important goal for acute care facilities. Implementing comprehensive programs such as the AGS CoCare: HELP can make a meaningful difference in both short- and long-term patient outcomes.

### 5.1. Recommendations

This feasibility study supports introducing smaller sections of larger protocols to make improvements in patient care. The high level of nurse buy-in present in this study has large implications for the ability to implement the Family HELP protocol at this site in the near future, with sustainability and continued use supported by inclusion of the task in the electronic health record charting. Exploration of the nurse perceived barriers to implementation is warranted and might include nursing and institutional level, and interdisciplinary members of the care coordination team. The potential also exists to expand by individual protocols to utilize the full AGS CoCare: HELP guidelines to reduce patient delirium further throughout the facility.

### 5.2. Sustainability

Suggested next steps include change sustaining addition of a timed task assignment populated to the Care Compass task bar paired with a formal roll out of the Family HELP protocol when family member visitation begins again. Eventual expansion might also include seeking direct feedback from patients and family members on their perceptions of the education, interventions, and outcomes.

## Figures and Tables

**Table 1:** Calgary Family Intervention Model (CFIM) Framework with Intervention Fit.

**Domain**	Interventions offered by the nurse: “Fit” or effectiveness
**Cognitive**	Teaching new actives and reasons
**Behavioral**	Encouraging behavioral changes through structured actions
**Affective**	Family HELP has demonstrated positive patient and family outcomes

## Data Availability

Data is contained within the article.

## References

[1] Devlin J.W., Skrobik Y., Gélinas C., Needham D.M., Slooter A.J.C., Pandharipande P.P., Watson P.L., Weinhouse G.L., Nunnally M.E., Rochwerg B. (2018). Clinical Practice Guidelines for the Prevention and Management of Pain, Agitation/Sedation, Delirium, Immobility, and Sleep Disruption in Adult Patients in the ICU. Crit. Care Med..

[2] CoCare HELP American Geriatrics Society. https://help.agscocare.org/.

[3] Morandi A., Piva S., Ely E.W., Myatra S.N., Salluh J.I., Amare D., Azoulay E., Bellelli G., Csomos A., Fan E. (2017). Worldwide Survey of the “Assessing Pain, Both Spontaneous Awakening and Breathing Trials, Choice of Drugs, Delirium Monitoring/Management, Early Exercise/Mobility, and Family Empowerment” (ABCDEF) Bundle. Crit. Care Med..

[4] Hshieh T.T., Yang T., Gartaganis S.L., Yue J., Inouye S.K. (2018). Hospital Elder Life Program: Systematic Review and Meta-analysis of Effectiveness. Am. J. Geriatr. Psychiatry.

[5] Furterer S.L. (2018). Applying Lean Six Sigma methods to reduce length of stay in a hospital’s emergency department. Qual. Eng..

[6] Rosenbloom-Brunton D.A., Henneman E.A., Inouye S.K. (2010). Feasibility of Family Participation in a Delirium Prevention Program for Hospitalized Older Adults. J. Gerontol. Nurs..

[7] Misto K. (2019). Family Perceptions of Family Nursing in a Magnet Institution During Acute Hospitalizations of Older Adult Patients. Clin. Nurs. Res..

[8] Smithburger P.L., Korenosk A.S., Kane-Gill S.L., Alexander S.A. (2017). Perceptions of family members, nurses, and physicians on in-volving patients’ families in delirium prevention. Crit. Care Nurse.

[9] Blevins C.S., DeGennaro R. (2018). Educational Intervention to Improve Delirium Recognition by Nurses. Am. J. Crit. Care.

[10] Grover S., Avasthi A. (2018). Clinical Practice Guidelines for Management of Delirium in Elderly. Indian J. Psychiatry.

[11] Lee H.W., Park Y., Jang E.J., Lee Y.J. (2019). Intensive care unit length of stay is reduced by protocolized family support intervention: A systematic review and meta-analysis. Intensiv. Care Med..

[12] Kang J., Lee M., Ko H., Kim S., Yun S., Jeong Y.J., Cho Y.S. (2018). Effect of nonpharmacological interventions for the prevention of delirium in the intensive care unit: A systematic review and meta-analysis. J. Crit. Care.

[13] Gorski S., Piotrowicz K., Rewiuk K., Halicka M., Kalwak W., Rybak P., Grodzicki T. (2017). Nonpharmacological Interventions Targeted at Delirium Risk Factors, Delivered by Trained Volunteers (Medical and Psychology Students), Reduced Need for Antipsychotic Medications and the Length of Hospital Stay in Aged Patients Admitted to an Acute Internal Medicine Ward: Pilot Study. BioMed Res. Int..

[14] Heim N., Van Stel H.F., Ettema R., Der Mast R.C.V., Inouye S.K., Schuurmans M.J. (2017). HELP! Problems in executing a pragmatic, randomized, stepped wedge trial on the Hospital Elder Life Program to prevent delirium in older patients. Trials.

[15] Zamoscik K., Godbold R., Freeman P. (2017). Intensive care nurses’ experiences and perceptions of delirium and delirium care. Intensive Crit. Care Nurs..

[16] Mitchell M.L., Kean S., Rattray J.E., Hull A.M., Davis C., Murfield J.E., Aitken L.M. (2017). A family intervention to reduce delirium in hospitalised ICU patients: A feasibility randomised controlled trial. Intensive Crit. Care Nurs..

[17] Rosgen B., Krewulak K., Ma D.D., Ely E.W., Davidson J.E., Stelfox H.T., Fiest K.M. (2018). Validation of Caregiver-Centered Delirium Detection Tools: A Systematic Review. J. Am. Geriatr. Soc..

[18] Cohen C., Pereira F., Kampel T., Bélanger L. (2019). Understanding the integration of family caregivers in delirium prevention care for hospitalized older adults: A case study protocol. J. Adv. Nurs..

[19] Leahey M., Wright L.M. (2016). Application of the Calgary Family Assessment and Intervention Models: Reflections on the Reciprocity Between the Personal and the Professional. J. Fam. Nurs..

[20] Kotfis K., Roberson S.W., Wilson J.E., Dabrowski W., Pun B.T., Ely E.W. (2020). COVID-19: ICU delirium management during SARS-CoV-2 pandemic. Crit. Care.

[21] Polit D.F., Beck C.T. (2017). Nursing Research: Generating and Assessing Evidence for Nursing Practice.

